# Structure- and Ligand-Based Virtual Screening Identifies New Scaffolds for Inhibitors of the Oncoprotein MDM2

**DOI:** 10.1371/journal.pone.0121424

**Published:** 2015-04-17

**Authors:** Douglas R. Houston, Li-Hsuan Yen, Simon Pettit, Malcolm D. Walkinshaw

**Affiliations:** 1 Institute of Structural and Molecular Biology, University of Edinburgh, Edinburgh, United Kingdom; 2 Selcia Limited, Fyfield Business & Research Park, Ongar, Essex, United Kingdom; University of Rome Tor Vergata, ITALY

## Abstract

A major challenge in the field of ligand discovery is to identify chemically useful fragments that can be developed into inhibitors of specific protein-protein interactions. Low molecular weight fragments (with molecular weight less than 250 Da) are likely to bind weakly to a protein’s surface. Here we use a new virtual screening procedure which uses a combination of similarity searching and docking to identify chemically tractable scaffolds that bind to the p53-interaction site of MDM2. The binding has been verified using capillary electrophoresis which has proven to be an excellent screening method for such small, weakly binding ligands.

## Introduction

Virtual screening (VS) to identify ligands that will interrupt protein-protein interactions remains challenging [1–3]. There are now a large number of VS success stories present in the literature, with targets as diverse as G-protein coupled receptors [4], enzymes such as angiotensin converting enzyme [5], zinc β-lactamase [6] and monoamine oxidase A [7], and Tat-TAR RNA Interactions [8] successfully targeted. However, most conventional VS approaches identify large hydrophobic molecules less suited to chemical modification; indeed it has been shown that docking programs tend to be biased in favour of larger molecules [9]. In addition, docking programs also struggle to accurately predict the binding modes of small fragment-like molecules [10]. Testing the predicted hits is also problematic for weak binding ligands, though SPR, NMR and ITC can be used if the ligands are sufficiently soluble. In this work we show capillary electrophoresis (CE) is a powerful technique with a number of advantages.

The target for this work is the p53 binding pocket of MDM2. The tumour suppressor p53 regulates the cell cycle through arresting growth and causing apoptosis in damaged or aberrant cells [11]. In unstressed cells, p53 is held at low levels to allow normal functions such as mitosis to continue. The E3 ligase MDM2 suppresses the activity of p53 via polyubiquitination and subsequent degradation by the proteasome [12–16]. Cancer cells have been shown to be particularly sensitive to restoration of p53 function, suggesting that inhibition of downregulators of its function should be a viable approach for the development of anticancer therapies [17–29].

**Fig 1:**
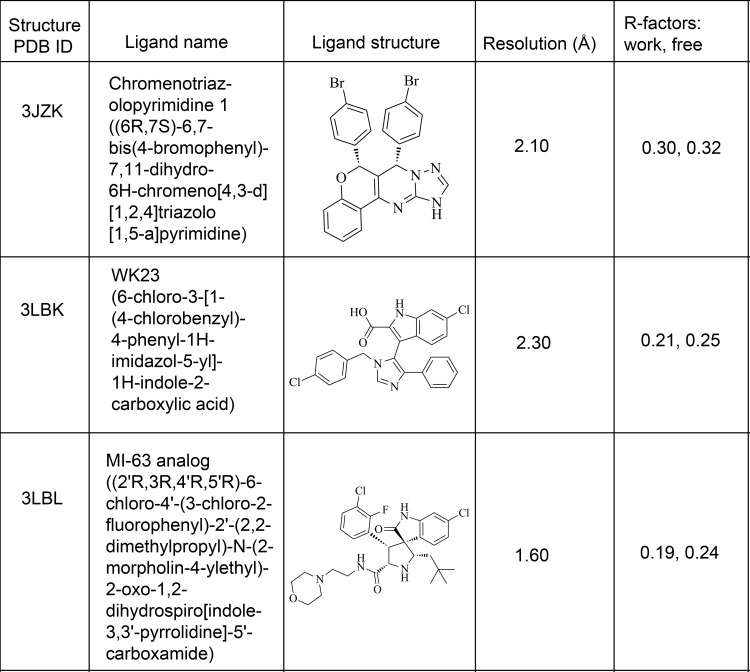
Crystal structures of MDM2 with bound small molecules.

There are several different classes of small molecule inhibitors of MDM2 that are able to interfere with MDM2-p53 binding with potency in the nM range (see **Fig 1** for details of a selection of these). One such molecule, named reactivation of p53 and induction of tumour cell apoptosis (RITA), has been shown to induce apoptosis in some cancer cell lines [30–32], although it may not be a classical MDM2-p53 interaction disruptor [33]. A second class of small molecules, the Nutlins, are high affinity inhibitors of MDM2 and induce activation of p53 by binding to the p53 binding pocket of MDM2 [34]. Spiro-oxindoles comprise a third class [25, 26, 35, 36]. In this work we identified a number of lead-like compounds, which led to the discovery of several fragments that provide new chemical scaffolds that could serve as the core of novel MDM2 inhibitor families.

## Materials and Methods

### Docking parameters and Control Experiments

Water molecules and other hetero atoms were removed from the structures and the program PDB2PQR 1.8 [37] was used to assign position-optimised hydrogen atoms, utilising the additional PropKa [38] algorithm with a pH of 7.4 to predict protonation states. The MGLTools 1.5.4 utility prepare_receptor4.py was used to assign Gasteiger charges to atoms. Hydrogen atoms were assigned to ligand structures using OpenBabel 2.3.1 [39], utilising the-p option to predict the protonation states of functional groups at pH 7.4. The MGLTools utility prepare_ligand4.py was used to assign Gasteiger charges and rotatable bonds. As Vina 1.1.2 [40] and Autodock 4.2.3 [41–43] both use the same. pdbqt format for their input, the same prepared files could be used for each. A grid box that encompassed the maximum dimensions of the ligand plus 12 Å in each direction was used. The starting translation and orientation of the ligand and the torsion angles of all rotatable bonds were set to random. The Autogrid grid point spacing was set at 0.2 Å. The Autodock parameter file specified 10 Lamarckian genetic algorithm runs, 2,000,000 energy evaluations and a population size of 300. Each docking program was used to automatically dock the ligand back into the p53 binding pocket of MDM2.

### Curation of the Virtual Chemical Library

The screening compound stock lists in SDF format of ChemBridge, Asinex, Maybridge, Enamine, LifeChemicals, Specs, InterBioScreen, ChemDiv and KeyOrganics were merged. Salts were stripped out using Sieve 3.1.0 [44], and duplicates removed using canonical SMILES string comparison via Open Babel 2.3.1. The supplied 2D coordinates were converted into 3D using Concord 4.08 [45]. Because the aim was to identify initial lead-like hits suitable for optimisation into more drug-like molecules, the virtual library was filtered according to Oprea "lead-like" rules (H-bond acceptors ≤ 9; H-bond donors ≤ 5; MW ≤460; cLogP ≥ -4.6 ≤ 4.2; cLogS ≥ -5; Number of rings ≤ 4; Number of rotatable bonds ≤ 9) [46]. This left 1,137,587 molecules. A multi-conformer version of this virtual library was produced using Multiconf-DOCK [47]; an average of 4.25 conformers per compound were generated depending on flexibility; this resulted in a virtual library containing a total of 4,840,093 conformers.

### Virtual Screening

An initial ligand-based pharmacophore screen was carried out. All ligands (both peptides and small molecules) present in the p53 binding pocket of MDM2 in all of the crystal structures in the PDB were used as search terms (S1 Table). The programs UFSRAT [48], ROCS [49] and a Wiener index similarity comparison algorithm available as part of EDULISS [50] were used to search the virtual library for molecules with different types of similarity to the known ligands. Specifically, UFSRAT (Ultra Fast Shape Recognition with Atom Types) is a shape recognition algorithm that takes into account atom types, which places importance not only on the space-filling properties of a small molecule, but also possible electrostatic interactions. An expansion of the validated [51] USR technique, the UFSRAT similarity calculation process consists of three steps: first, shape and atom property descriptors are calculated for each molecule; second, the descriptors are compared using a scoring function, and finally, similar molecules are ranked by score. The UFSRAT search of the multiconformer library returned 9,999 molecules.

ROCS (Rapid Overlay of Chemical Structures) is also a shape matching algorithm but uses a smooth Gaussian function to represent the molecular volume, analytically optimizing the volume intersection of the two molecules being compared [52]. The shape comparison method uses “shape multipoles” that can be used to describe the inherent shape of molecules. This is coupled with chemistry matching based on a user-definable chemical force-field. The ROCS search of the multiconformer library returned 10,021 molecules.

Wiener index comparison is a well-known and long-established method in chemical graph theory that can be used to measure molecular similarity [53]. A type of molecular descriptor, the Wiener index (also known as Wiener number) is a topological index of a molecule, defined as the sum of the lengths of the shortest paths between all pairs of vertices in the chemical graph representing the non-hydrogen atoms in the molecule [54]. The calculation of this index by EDULISS 2.0 deduces the path number and the polarity number of a compound. The polarity number is defined as the number of pairs of atoms which are separated by three bonds. The query and library compound comparison is based on Euclidean distance, i.e. the shorter the distance between the indices of query and library molecule, the more the connectivity of their bonds is similar. The EDULISS search returned 4,545 molecules.

In addition, the rigid-body docking program LIDAEUS [55] was used to dock the conformer virtual library into the p53 binding site of the MDM2 crystal structure. The results were ranked according to LIDAEUS score, the top 65,906 compounds from this list merged with the results from the ligand-based methods described above, and the duplicates removed. This resulted in 79,611 unique molecules which were then docked into MDM2 using Vina. Docked poses were scored using both Vina’s internal scoring algorithm and X-Score 1.2 [56]; these scores were used via a “rank-by-rank” consensus scheme [57] to create a ranked list. The top 4,469 compounds were then docked using Autodock. Predicted binding poses were also scored using DrugScore 1.2 [58]. The addition of DrugScore and X-Score to the scoring scheme was prompted by the results of several groups which have shown that these programs are among the most accurate in predicting affinity [59, 60], and that the use of several scoring algorithms in a "consensus scoring" scheme generally produces better results, both in virtual screening arena [10, 59–65] and in related fields [66–68]. Indeed, information fusion has been shown to improve performance in a broad range of human endeavours too numerous to exhaustively list (a few interesting examples include competitive chess [69], medical diagnosis [70], biometric recognition systems [71] and battlefield target identification [72]). A final ranked list was prepared via a rank-by-rank scheme, taking the Vina, Autodock, X-Score and DrugScore scores into account.

### Capillary Electrophoresis (CE)

Capillary electrophoresis (CE) is an analytical technique used to study the interaction between biomacromolecules [73, 74]. In CE, electrophoresis is carried out in the interior of a narrow capillary. The technique separates ions based on their electrophoretic mobility with the use of applied voltage. The electrophoretic mobility is dependent on the charge of the molecule, the viscosity and the molecular size. CE is performed using a high voltage to produce a high resolution profile with a short migration time to separate numerous analytes. CE possesses several advantages over other common techniques such as Surface Plasmon Resonance (SPR), Nuclear Magnetic Resonance (NMR) or Isothermal Calorimetry (ITC). For example, there is no protein size limit, the protein does not need to be modified or tethered, and the technique results in little sample consumption. The assay provides a high sensitivity through the use of a Laser Induced Fluorescence (LIF) detector to enable the detection of weak binding fragments. This enables the interaction assay to be performed with a low labelled probe concentration and allows compounds to be screened at low concentrations. A competitive CE assay was used to detect competition between compounds and fluorescently labelled p53 peptide (p53-F) in MDM2-N/p53 binding site. The injection sample contained carboxyfluorescein (internal standard (10pM)) and p53-F (500pM) in TAPS/Tris (1mM) pH 8.0, DTT (1mM). The separation sample contained MDM2-N (160nM), test compound (300μM), TAPS/Tris (200mM) pH 8.0, DMSO (1% f/c) and glycerol (0.25%). Compounds were screened at 300μM concentration as the initial screening concentration, depending on the compound solubility. After injection of the sample, a voltage of 10kV was applied and separation was carried out for 6.5min. The migration time of the p53-F peak was detected by laser induced fluorescence (LIF) detector and the inhibition (%) was calculated using the equation below.

%Inhibition= 100(TH-TL)/(TH-TR)

Where: T_H_ is migration time of p53-F peak in the presence of MDM2-N.

T_R_ is the migration time of p53-F peak.

T_L_ is the migration time of p53-F peak in the presence of MDM2-N + compound.

### Fluorescence polarization (FP) assay

Fluorescence polarization experiments were performed with a LJL Biosystems Analyst multi-plate reader in 50mM HEPES pH 7.5, 100mM NaCl, 1mM DTT. Fluorescein labelled p53 peptide (p53-F) was synthesised by Biomatiks. The excitation and emission wavelengths were set to 490 and 525nm. The competition experiments were carried out by mixing MDM2-N (500nM), p53-F (2nM) and test compound in black NBS plate. Compounds were screened at 700μM concentration as the initial screening concentration, depending on the compound solubility. The inhibition (%) was calculated using the equation below.

%Inhibition=100(AF-AL)/(AF-AU)

Where: A_F_ is the anisotropy of fully bound p53-F.

A_U_ is the anisotropy of unbound p53-F.

A_L_ is the anisotropy of p53-F in the presence of MDM2-N + compound.

### Ethics Statement

No approval from any ethics committees was required as no animal, human, cell line or field sampling experiments took place.

## Results

### Redocking Control Experiment

**Fig 2:**
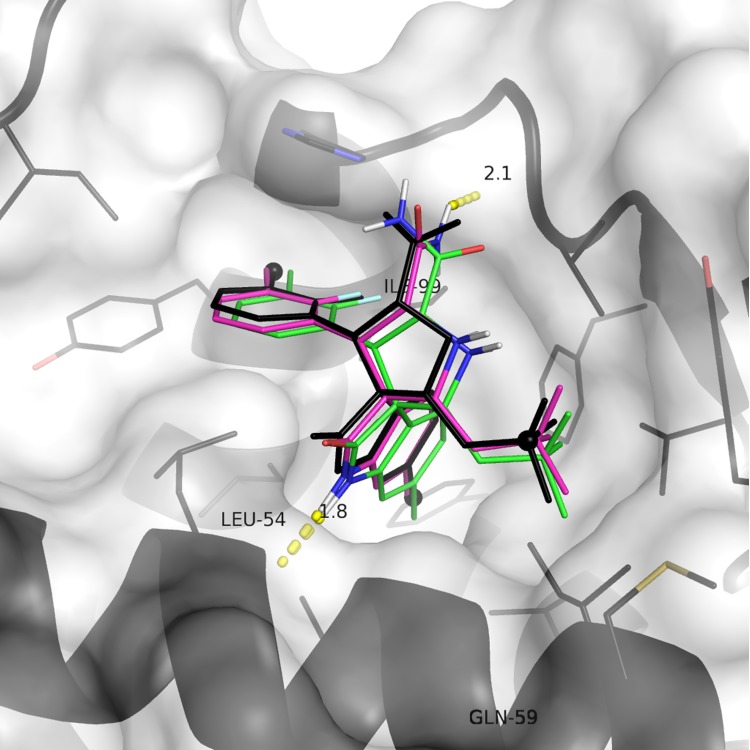
Control docking results. Crystallographic pose is coloured black, intermolecular hydrogen bonds are shown as orange dashes, Autodock result is red, Vina result is magenta. Pharmacaphore filter points are shown as black spheres. The protein is represented as a white transparent surface and cartoon secondary structure; residue side chains within 5 Å of the ligand are shown as black lines.

The structure of MDM2 in complex with an MI-63 analogue was used for the docking studies (PDB 3LBL, Fig 1). Its high resolution (1.60 Å), good R-factors (R = 0.193, R_free_ = 0.236) and the small molecule ligand-binding conformation of the protein made it ideal for this purpose. The ligand present in the structure was redocked to verify that the docking programs used for the virtual screening were successfully able to correctly predict its binding conformation. Autodock successfully reproduced the crystallographic binding mode of the ligand, the result being within an RMSD of 0.83 Å. Vina also correctly predicted the binding pose of the ligand, with an RMSD of 0.67 Å. Autodock predicted the free energy of binding to be -9.4 kcal/mol, Vina predicted -9.8 kcal/mol. These are both roughly equivalent to a Ki around the 100 nM mark, and are remarkably close to the measured Ki of 36 nM [36]. Fig 2 shows a superposition of the docking results with the crystal structure.

### Virtual Screening Results

**Fig 3:**
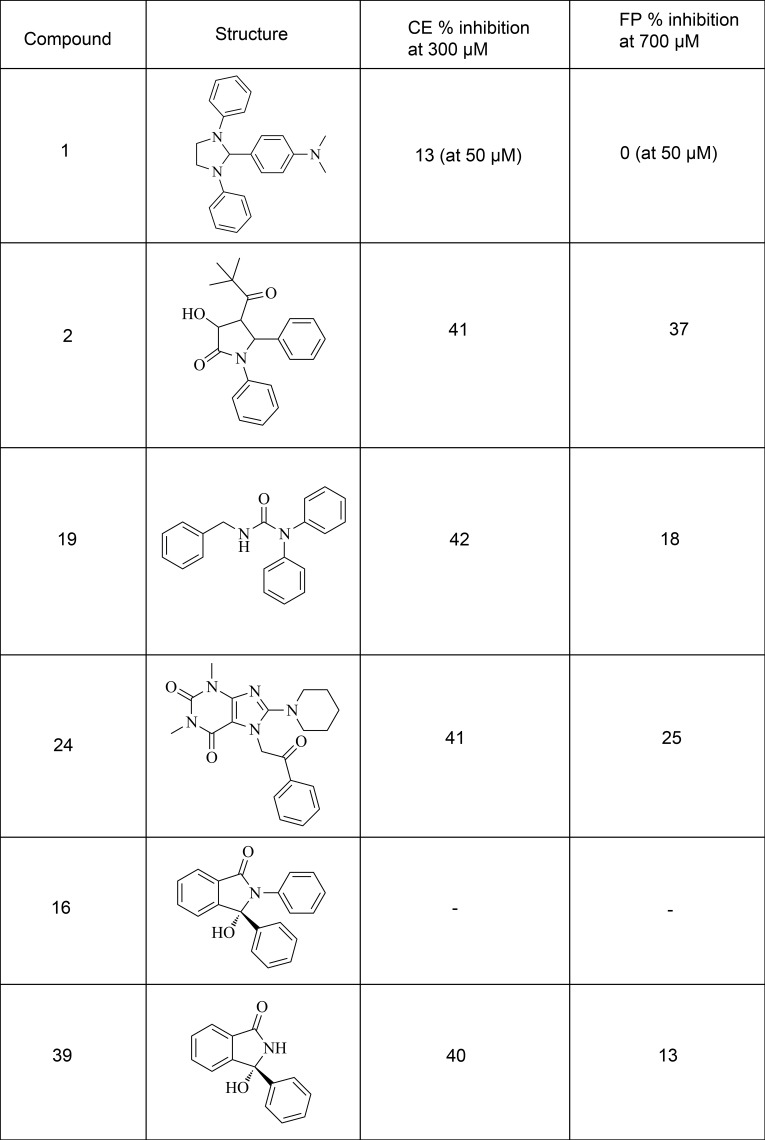
Structural formulae of the 5 hits identified through the initial virtual screen.

An initial ligand-based pharmacophore screen was carried out. A total of 23,588 compounds were returned. Vina and then Autodock were used to dock the top hits from LIDAEUS. A simple pharmacophore filter was designed by identifying the main features of the MI-63-analog ligand that are involved in interactions with MDM2 (Fig 2). This resulted in a final ranked list of 2120 molecules; the predicted binding modes of the top 266 were inspected manually for final selection. 38 of these were chosen for acquisition and assay. S2 Table lists their details. Compound 16 was found to be unavailable for purchase, therefore the most similar compound in stock was selected as a substitute (39, Fig 3)

### CE binding Assay results

**Fig 4:**
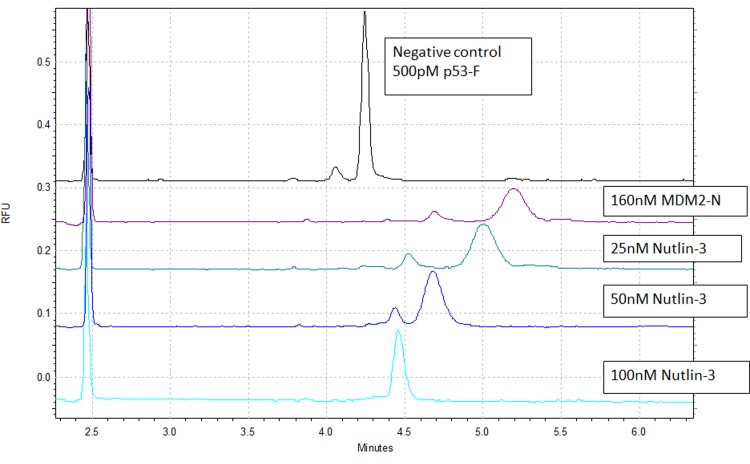
Nutlin-3 titration. As the Nutlin-3 concentration increases the peak shifted back to p53-F control peak. Top trace is the p53-F alone followed by p53-F with MDM2-N. Nutlin-3 was then added into the sample with increasing concentration.

Racemic Nutlin-3 was used to confirm if the p53-F/MDM2-N peak shifted to its original “apo” position in the presence of a known inhibitor. Upon adding Nutlin-3 (100nM), the peak shifted back to the p53-F peak position with a change in peak shape indicating the competition between p53-F and Nutlin-3 in the MDM2-N binding site (Fig 4, lower trace).

**Fig 5:**
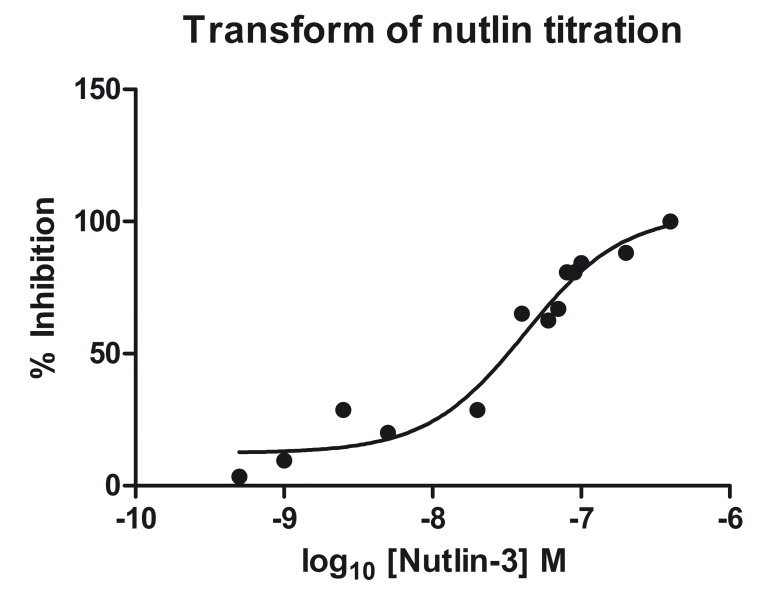
Graph showing inhibition (%) plotted against Nutlin-3 concentration. From the data an IC50 of 37.3nM was determined.

With additional titration, the IC_50_ of Nutlin-3 could be calculated. Nutlin-3 was titrated from 400 nM to 0.5 nM and % inhibition was calculated from the migration time of Nutlin-3 (Fig 5). The % inhibition was fitted against Nutlin-3 concentration in log scale in GraphPad Prism and IC_50_ was calculated to be 37.50 nM. The calculated IC_50_ is similar to the literature value for racemic Nutlin-3 of 100 nM [34]. This confirms that CE is able to recognise inhibitor binding and able to calculate an accurate IC_50_.

### FP assay results

**Fig 6:**
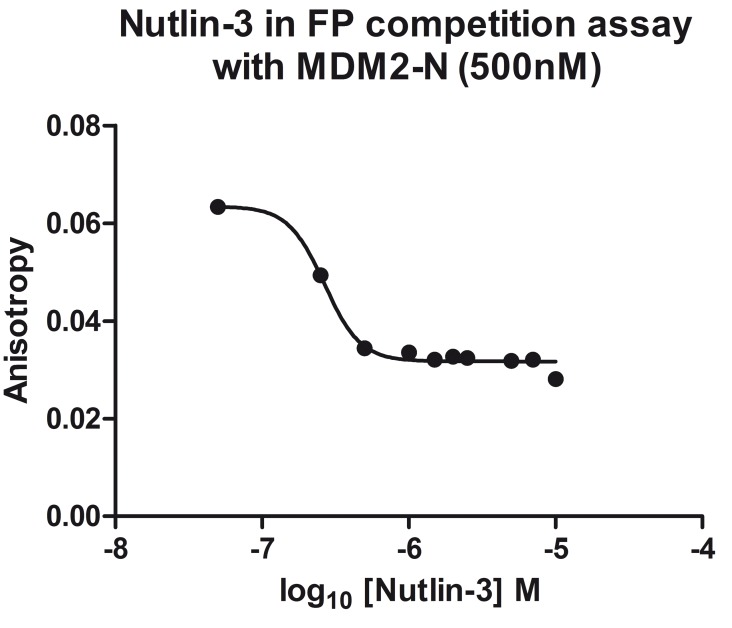
Competition of Nutlin-3 with p53-F for MDM2-N measured by FP assay. The graph shows anisotropy plotted against Nutlin-3 concentration. An IC_50_ of 266 nM was determined.

The FP competition experiment was carried out to test whether the FP assay was suitable for identifying small molecule inhibitors for MDM2-N. A dose response experiment was performed using Nutlin-3 from 0.02 μM to 10 μM. MDM2-N concentration was held constant at 500 nM and p53-F was 2 nM in the mixture. The anisotropy was fitted against Nutlin-3 concentration in GraphPad Prism and IC_50_ was calculated to be 266 nM (Fig 6). Five of the virtual screening compounds were identified to be active in both CE and FP assay (Fig 3).

### Analogue exploration

**Fig 7:**
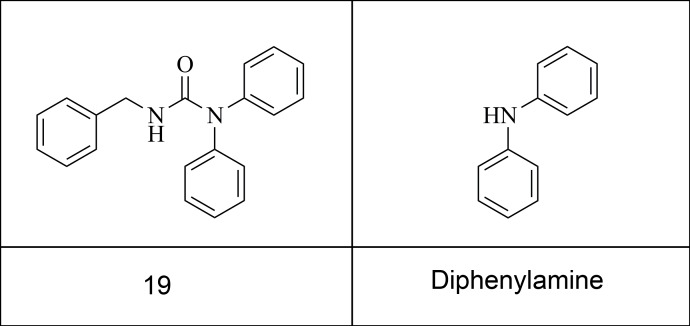
Diphenylamine fragment based on the virtual screening hit 19.

These initial virtual screening hits, although active, exhibited high molecular weight and low solubility. Therefore it was determined that smaller molecular weight fragments with higher solubility should be identified. Analogues of the virtual screening hit 19 were further explored because the structure was novel and different from known inhibitors. 19 was further explored using the Selcia chemical store substructure search tool in the Selcia compound library. Compounds containing a similar motif as 19 (Fig 7) were tested in the CE and FP assay.

**Fig 8:**
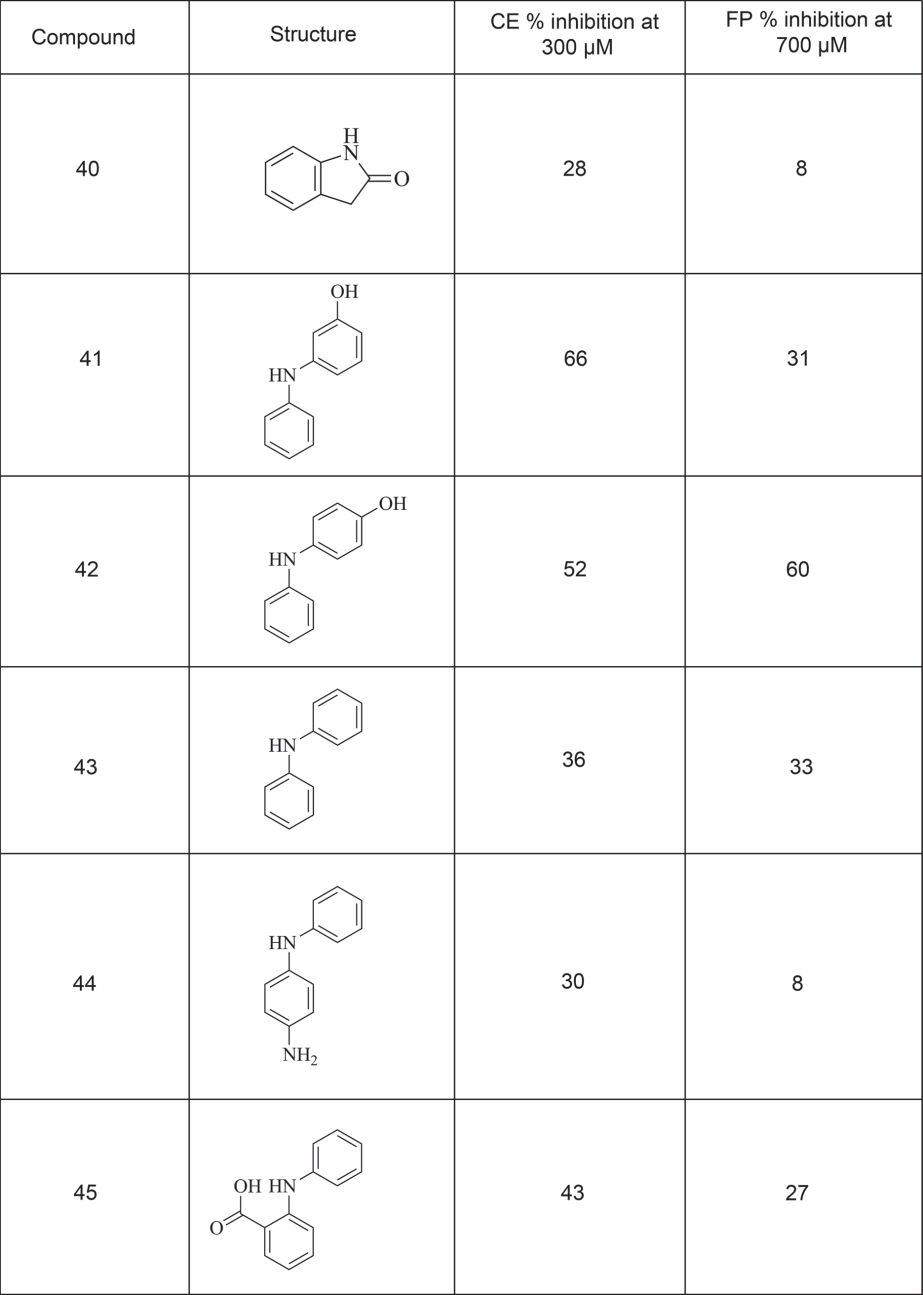
Further exploration of analogues of virtual screening hit 19. 6 analogues selected on the basis of the hits showed inhibition in both the CE and FP assays.

A total of 5 of the 38 compounds acquired on the basis of the analogue exploration showed inhibition of MDM2 in the CE assay at 300 μM (Fig 8). An additional compound was tested at only 50 μM due to solubility issues and this also showed inhibition. Of these 6 compounds, 4 showed greater than 10% inhibition of MDM2 in the orthogonal FP assay at 700 μM (Fig 8). The less soluble compound again could only be tested at 50 μM and showed no inhibition in the FP assay at this concentration.

## Discussion

Inspection of the output from the initial ligand-based pharmacophore screen found that compounds 1, 2, 16 and 19 were present in the output from the ROCS 3D similarity search. Compounds 16, 19 and 24 were present in the output from the UFSRAT 3D similarity search. Only 16 was present in the EDULISS 2D similarity search output. None of the compounds that exhibited activity in both assays were present in the LIDAEUS rigid-body docking output. Compounds 1, 2, 16 and 19 had been ranked most highly by the X-Score scoring algorithm, with 24 selected by Vina’s scoring algorithm. Vina has previously been shown to perform well in predicting the binding modes and relative affinities of MDM2 ligands [51] such as MI-63 and Telmisartan, which have been included in S2 Table for comparison. We also found that we were able to reproduce the predicted binding modes of MI-63 and Telmisartan as described in Patil et al. using Vina (Fig 2 and S1 Fig). In addition, the scoring algorithms chosen would have successfully identified both of these known high-affinity actives (S2 Table).

All of the actives contain either chiral centres in their central scaffolds or rotatable bonds positioned such that, when adopting their predicted binding conformations, they can substantially occupy all three dimensions in space. This 3D characteristic has recently been identified as important both for MDM2 inhibitors and for protein-protein interaction inhibitors in general [75, 76].

Compounds 1 and 2 both contain central imidazole or pyrrole groups to which three hydrophobic groups are attached (Fig 3). These dock into the three pockets of MDM2’s binding site, into which the Phe, Trp and Leu side chains of p53 bind. Of the 38 compounds tested, Compound 36 and Compound 38 appear structurally similar to these hits yet were inactive. However, both of these inactive compounds are planar whereas the two active compounds possess groups that project out from the plane of the imidazole group. The importance if this structural feature is confirmed when these compounds are compared to the cis-imidazolidines, for example Nutlin-3, in which both of its chlorobenzene groups project out of the plane of the molecule. RG7388 and RG7112 are molecules of this class of central imidazole or pyrrole scaffold that have very recently been identified and entered into clinical trials [77, 78]. Other pyrrole-containing MDM2 inhibitors have also been described recently [79]. AMG 232 is a somewhat related class of molecule from a pharmacophoric perspective, as although its central ring is a 6-membered piperidine group this still act as the nonplanar scaffold which positions the three attached protein-interacting hydrophobic groups. It too was recently identified and has entered clinical trials [80].

We were unable to test the isoindolinone 16 as although it was present in our virtual chemical library, which is based on the stock lists of various commercial compound suppliers, it was unavailable for purchase at the time of enquiry. Therefore, the most similar compound available was selected instead. Compound 39 constitutes a substructure of 16 (Fig 3). On identification of its activity in the assays a literature search revealed that this moiety has already been found to serve well as a scaffold for the design of MDM2 inhibitors [81].

**Fig 9:**
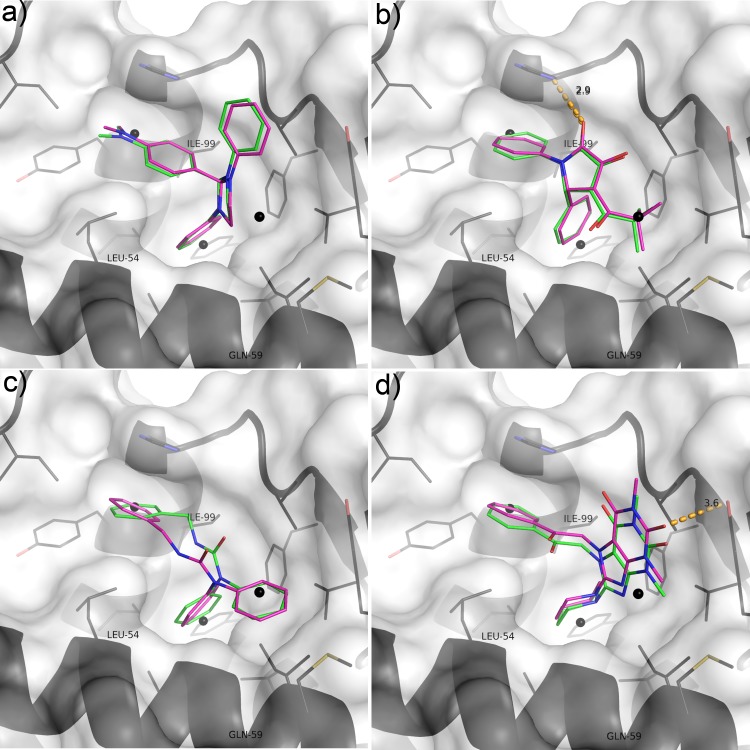
Lowest energy Vina (magenta) and most populous cluster Autodock (green) docking poses of the active compounds. The pharmacophore filter points are shown as black spheres. MDM2 is shown as a white transparent surface representation with the backbone visible as black secondary structure. Side chains of residues that line the active site are shown as black sticks. A) Compound 1; B) Compound 2; C) Compound 19 D) Compound 24

Compound 24 appears more novel, possessing little similarity with the current classes of known MDM2 small molecule inhibitors (Fig 1 and Fig 3). Three similar compounds were inactive, one of which differed by only one methyl group on the piperidine moiety. Compound 26 contains a 4-methylpiperidine, Compound 25 a 2,4-dimethylimidazole, and Compound 32 a cycloheptane group at this location, all of which are larger and, according to the docking results, project deeper into the p53 Trp pocket of MDM2 (Fig 9). The docking and assay results combined suggest a strict size limit on the length of the moiety that this pocket can accommodate.

**Fig 10:**
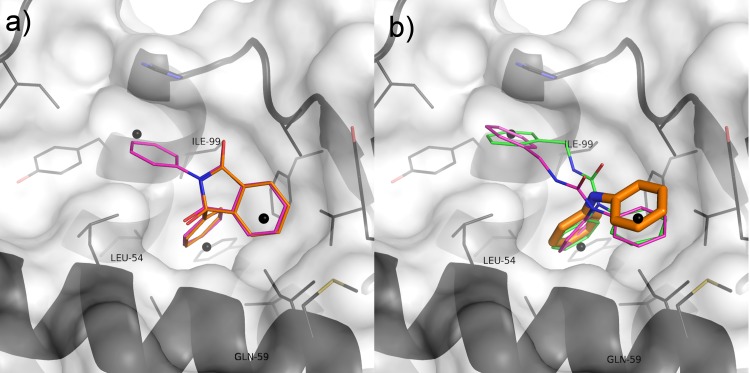
A) Vina docking poses of Compound 16 (magenta) and Compound 39 (orange); B) Vina docking poses of Compound 19 (magenta) and diphenylamine (orange).

Compound 19 is also novel compared to known inhibitors. It was also the only compound tested that contained a diphenylamine group. Seven of the compounds tested contain diphenylmethane groups, which are superficially similar, but none were active. This prompted an investigation of the contribution the diphenylamine group makes to binding. Several diphenylamine-containing fragments were tested, five of which showed inhibition in both assays (Fig 8). Docking these fragments suggests that they adopt the same binding conformation as the equivalent group in 19 (Fig 10 and S1 Fig and S3 Table). The predicted binding mode suggests that the central NH group does not take part in any hydrogen bonding with the protein, therefore it appears likely that it is the difference in configuration between the secondary carbon and the amine group (pyramidal vs. planar) that is responsible for the difference in activity. However, analysis of additional low energy binding modes for these molecules suggests that they may adopt alternate binding positions in the pocket (S1 Fig and S3 Table). Indeed it is to be expected that the limited number of interactions small fragments can make with a pocket bestows them with pharmacophores of limited structural specificity and thus a propensity to promiscuity. Both in silico studies and in vitro assay experiments confirm this [10, 82–84]. This is borne out when a comparison of ΔG is made between Vina's top docking solutions; this indicates that larger molecules tend to exhibit clearer docking solutions than fragments (S4 Table). Telmisartan appears to be the exception to this, likely due to the relatively large number of rotatable torsions making this compound more flexible, and multiplying the number of ways it can potentially fit the binding site. MI-63 has only four rotatable bonds, whereas Telmisartan has seven; this makes MI-63 more likely than Telmisartan to have only one way it can fit the pocket with significantly lower energy, as reflected both in the shape of its graph in S4 Table and the consensus exhibited between consecutive Autodock docking attempts (S3 Table).

## Conclusion

Although the library used for the virtual screening had been filtered according to "lead-like" rules (because the initial aim was to identify compounds suitable for optimisation into more drug-like molecules), subsequent investigation found that some of the compounds contained a fragment that also inhibits MDM2. The activity against MDM2 of diphenylamine, identified here though exploration of virtual screen hits, has not been previously identified. None of the previously known small molecule inhibitors of MDM2 contain this group. Its relative potency despite its small size means that it possesses good ligand efficiency, a characteristic that is highly desirable in an inhibitor scaffold. As such it could form a starting point for a series of diphenylamine based inhibitors that form interactions with a greater portion of MDM2’s p53 binding site, for which full binding curves to determine K_i_s will be necessary to perform a complete SAR analysis.

The virtual screening method used to discover these novel compounds is generally applicable to any target for which an atomic-resolution structure is known; therefore further inhibitor discovery using this technique is being pursued. It was noted that the 3D ligand-based comparison techniques identified the majority of the actives, not the 2D comparison or the 3D rigid-body docking methods. In addition, only scoring by X-Score and Vina (methods which are somewhat related [40]) brought these compounds to the top of the ranked list. However the sample size of four actives in total is too small to draw any conclusions regarding which computational or ranking method was superior.

## Supporting Information

S1 Fig Docking poses for the diphenylamine-based fragments; Vina (magenta) and Autodock (green) are shown as sticks with polar hydrogens visible; predicted hydrogen bonds are depicted as yellow dashed lines; a) compound 43; b) compound 45; c) compound 41; d) compound 42; e) compound 44 (TIF)

S1 Table Structural formulae of ligands in complex with MDM2 used for the ligand-based virtual screening. (DOCX)

S2 Table Virtual screening statistics of compounds selected for assay. Rank scores within the various ranking schemes are indicated, noninteger rank scores indicate positions tied between an even number of compounds; positions were determined for all compounds for which Autodock and Vina both predicted similar binding modes; this numbered 2,120 compounds. For those compounds found to be active, the scoring algorithm that ranked them highest is indicated by highlighting in the rank position in yellow. *Compounds found to exhibit activity in both the CE and FP assays. ^**†**^Compound not in stock at time of acquisition and substituted by the similar compound **39**. Results for the known inhibitors MI-63 and Telmisartan are included for comparison.
(DOCX)

S3 Table Summary of docking energies of fragment low energy binding modes versus controls. *Molecules that passed the "consensus docking" filter criteria. ^†^Molecules for which Autodock finds only one cluster of docking solutions—experience suggests that these tend to be more reliable predictions of binding mode.
(DOCX)

S4 Table Vina predicted ΔG for each of its top 9 docking solutions. Larger molecules (with the exception of the flexible Telmisartan) tend to exhibit clearer solutions than fragments.
(DOCX)

S5 Table Summary of the solubilities of the fragments in assay buffer. (DOCX)
