# Supplementary figures and images for: Structure- and Ligand-Based Virtual Screening Identifies New Scaffolds for Inhibitors of the Oncoprotein MDM2
Source: PLoS One. 2015 Apr 17;10(4):e0121424. doi: 10.1371/journal.pone.0121424 (PMC4401541; doi:10.1371/journal.pone.0121424)

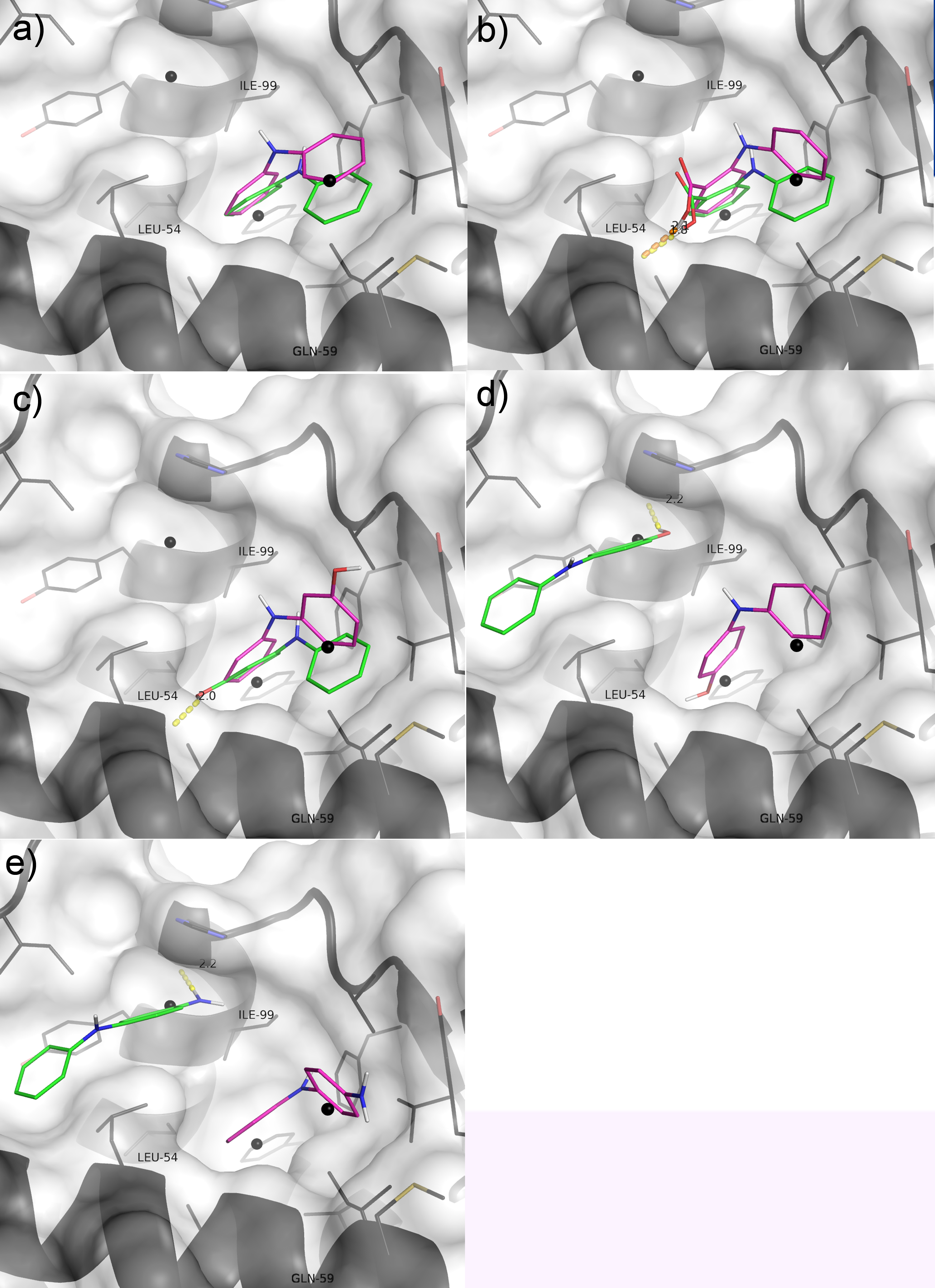

Supplement: S1 Fig — (TIF) [file pone.0121424.s001.tif]
