# Supplementary material for: Structure- and Ligand-Based Virtual Screening Identifies New Scaffolds for Inhibitors of the Oncoprotein MDM2
Source: PLoS One. 2015 Apr 17;10(4):e0121424. doi: 10.1371/journal.pone.0121424 (PMC4401541; doi:10.1371/journal.pone.0121424)
Supplement: S1 Table — (DOCX) [file pone.0121424.s002.docx]

S1 Table. Structural formulae of ligands in complex with MDM2 used for the ligand-based virtual screening.

| 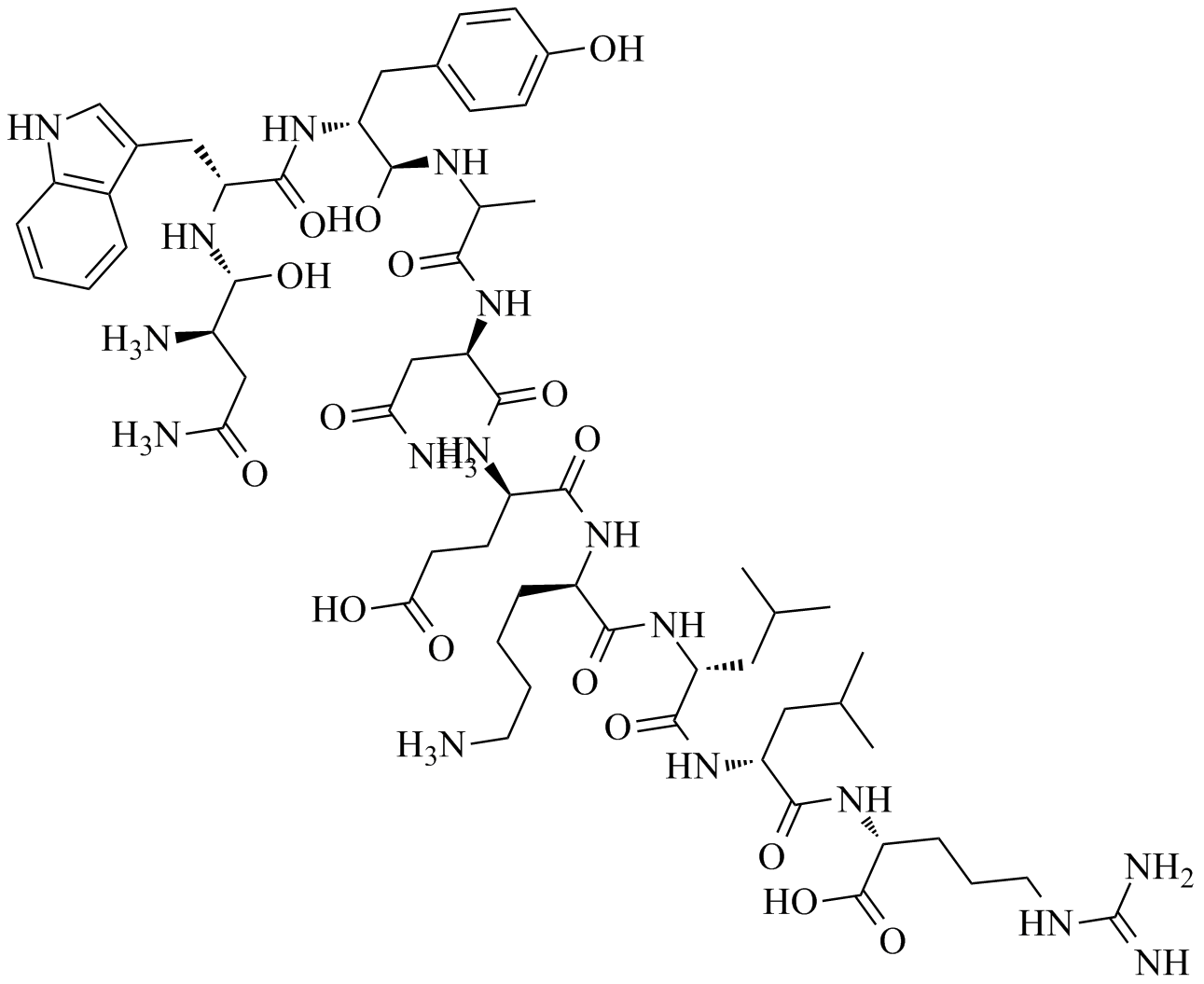  DPMI-alpha | 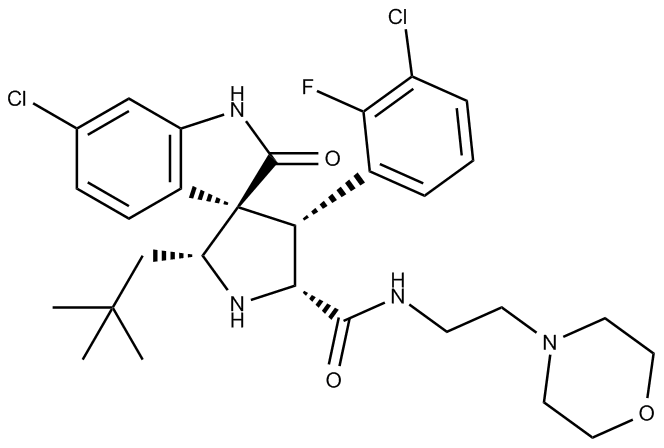  Mi63 | 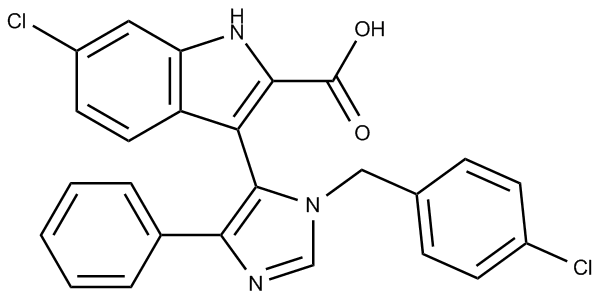  WK23 |
| --- | --- | --- |
| 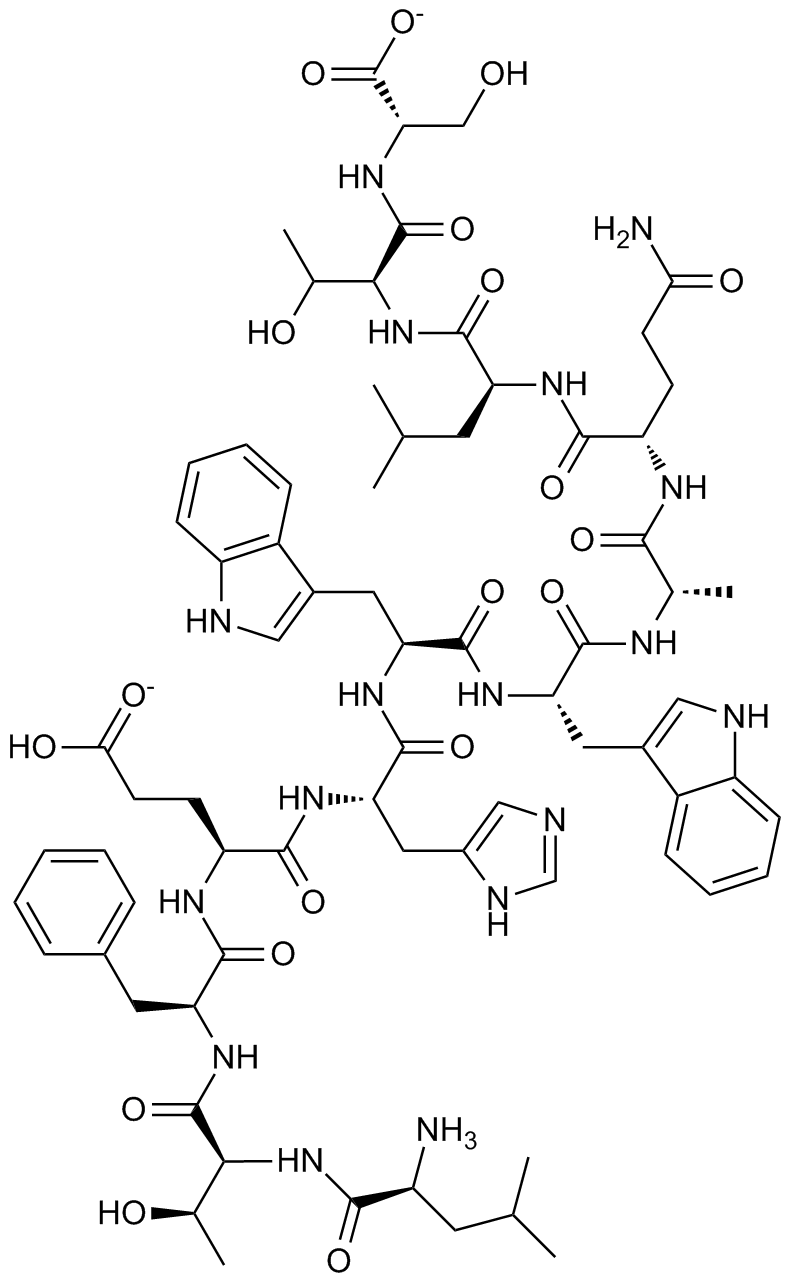  pDI6W | 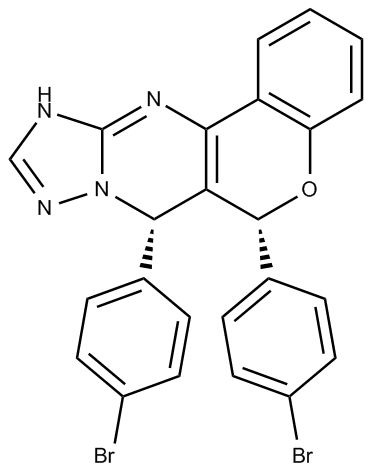  Chromenotriazolopyrimidine | 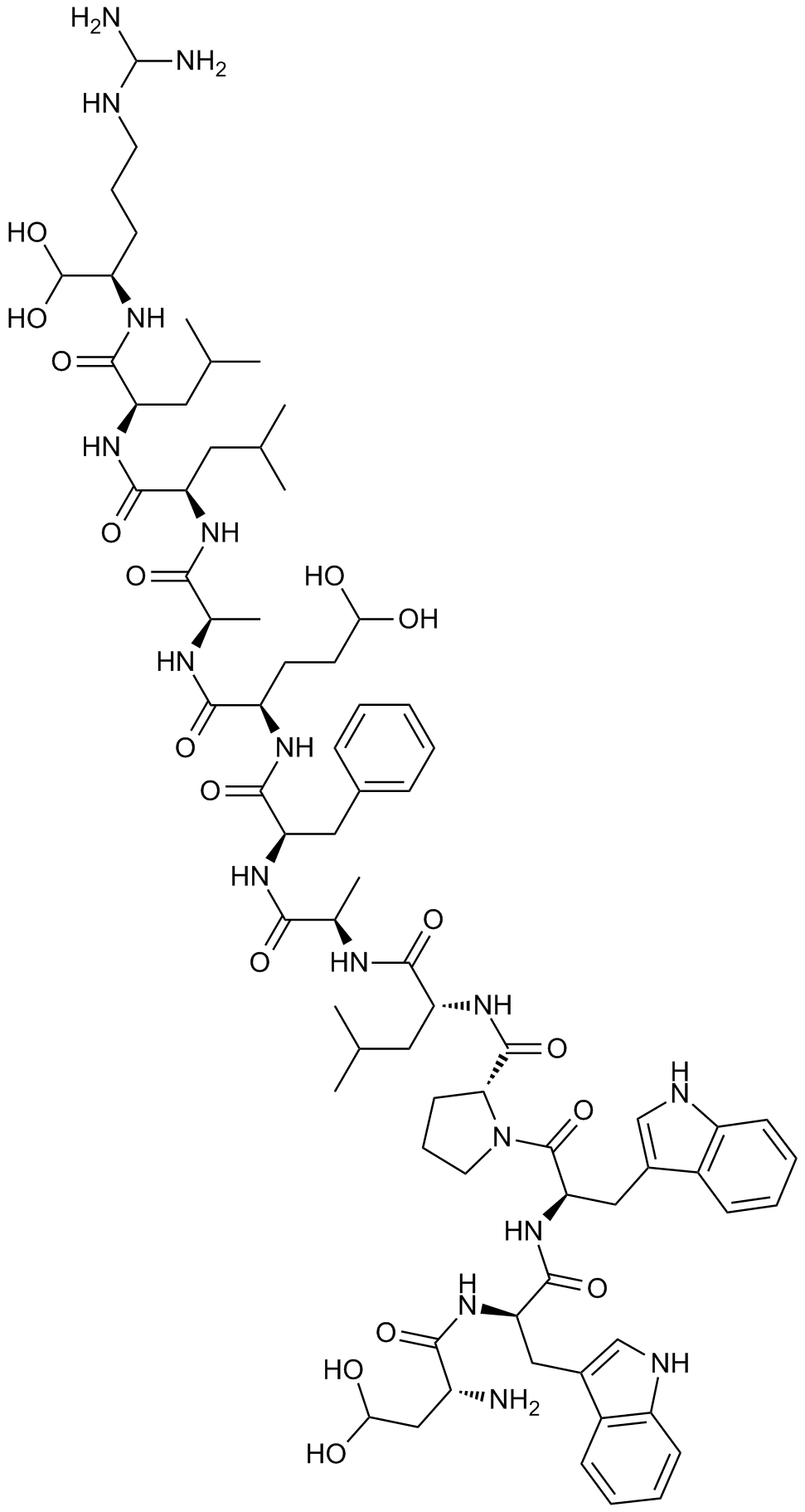  DPMI-gamma |
| 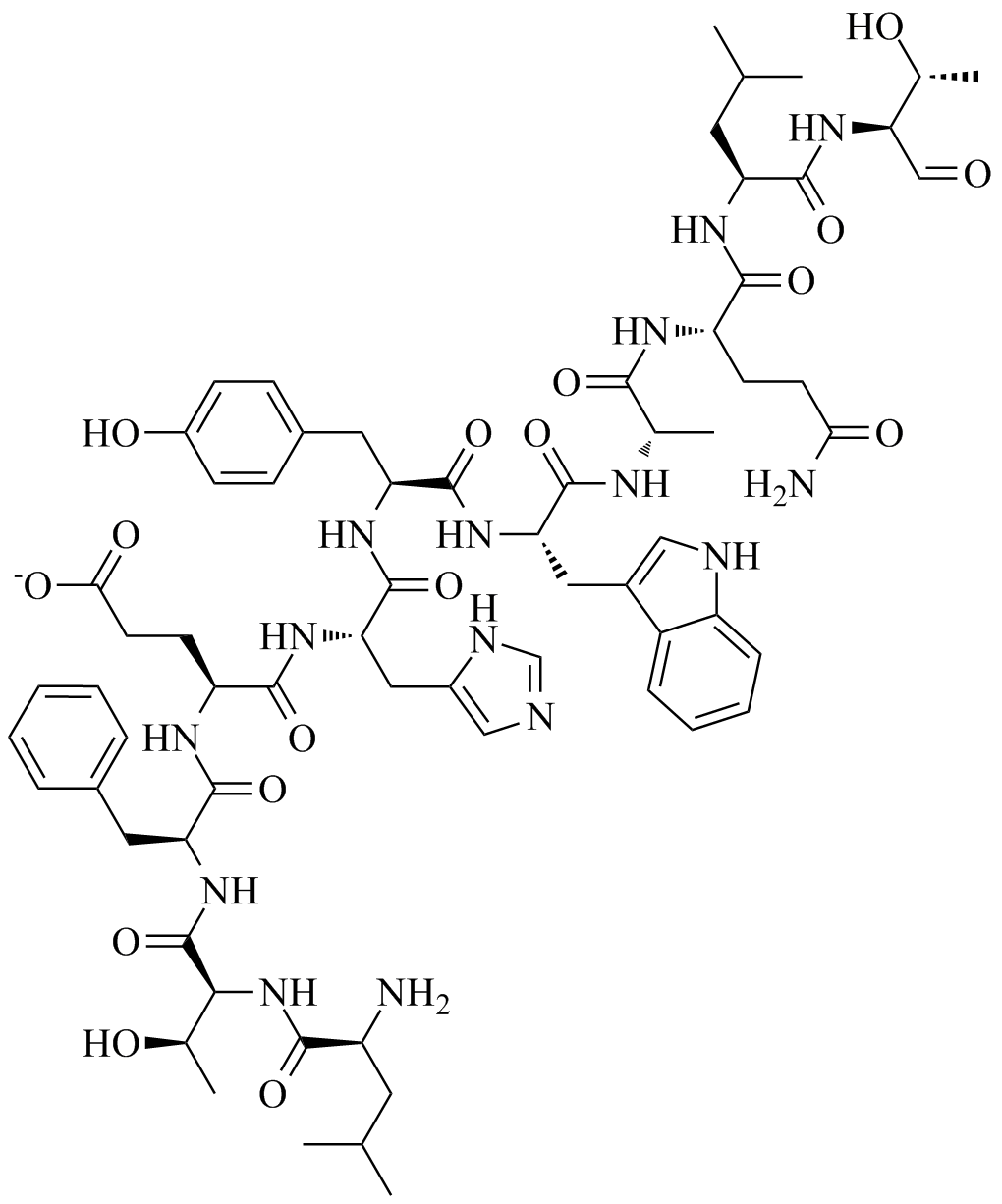  P4 peptide | 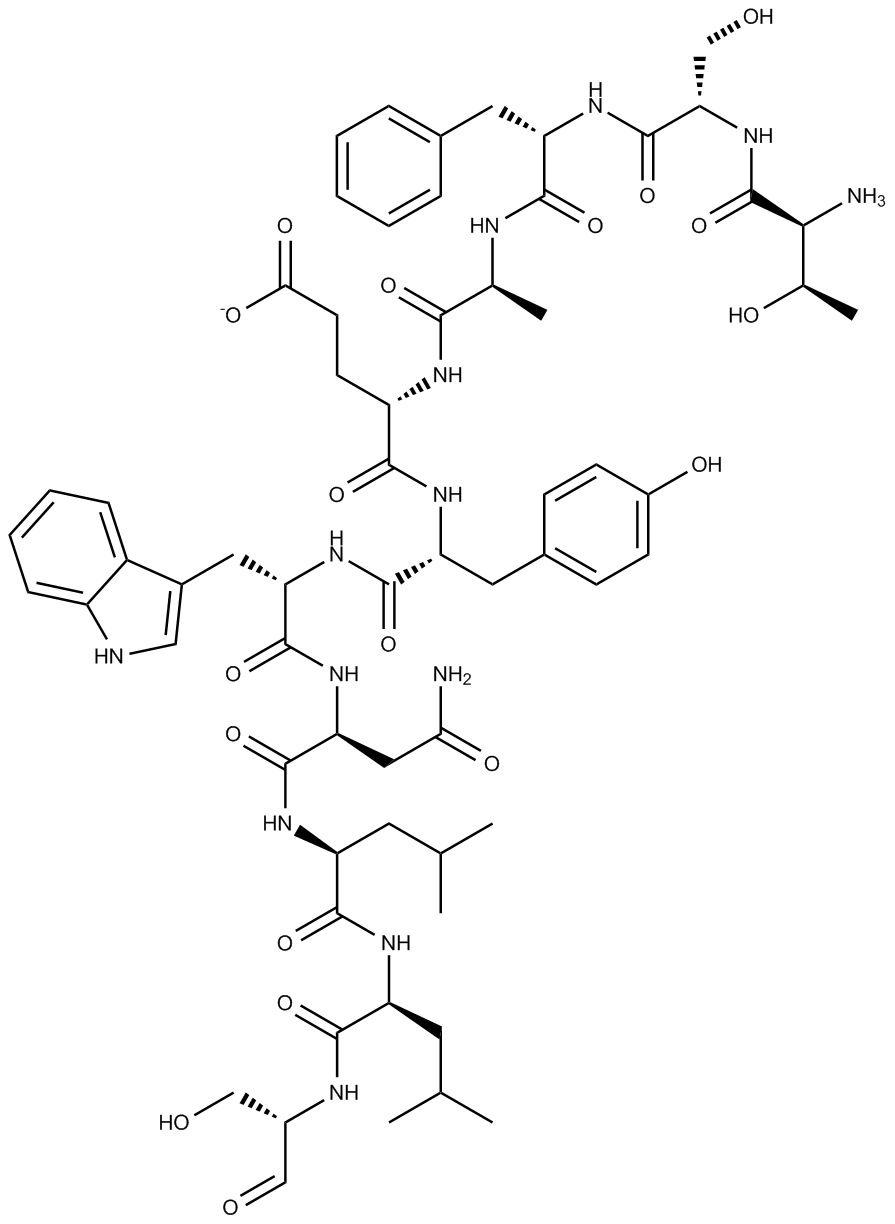  PMI | 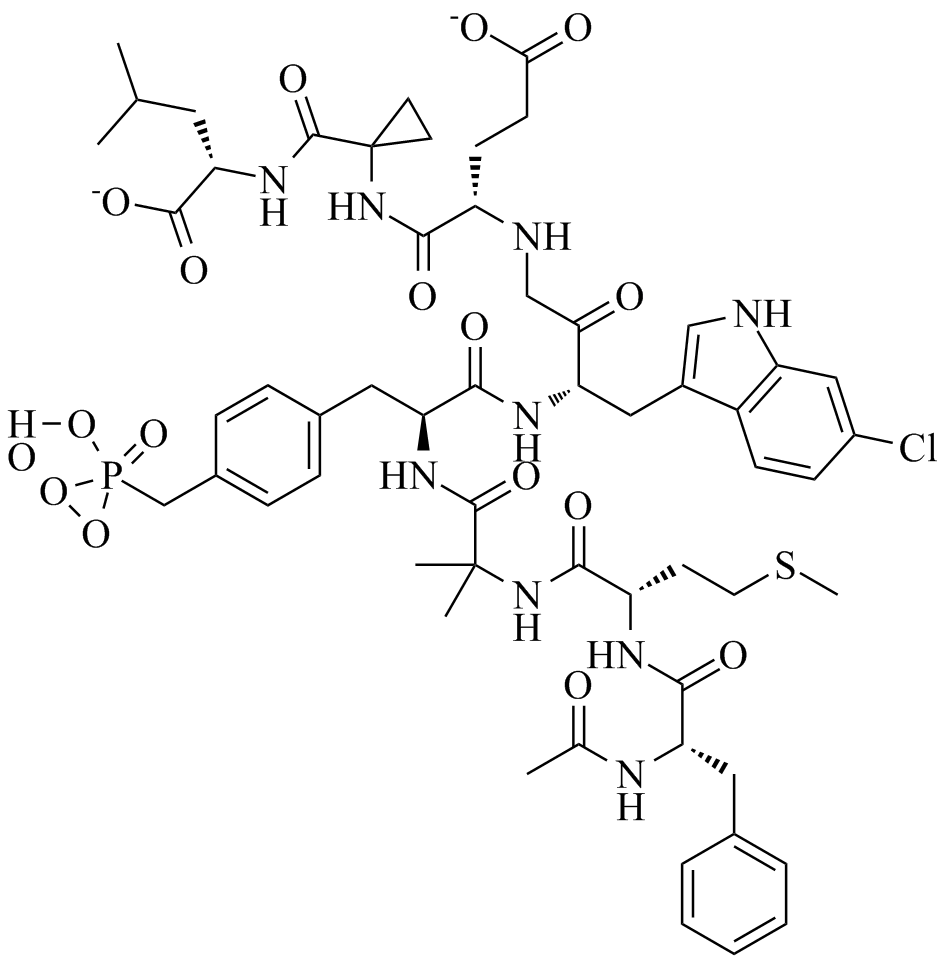  8-mer peptide 2 |
| 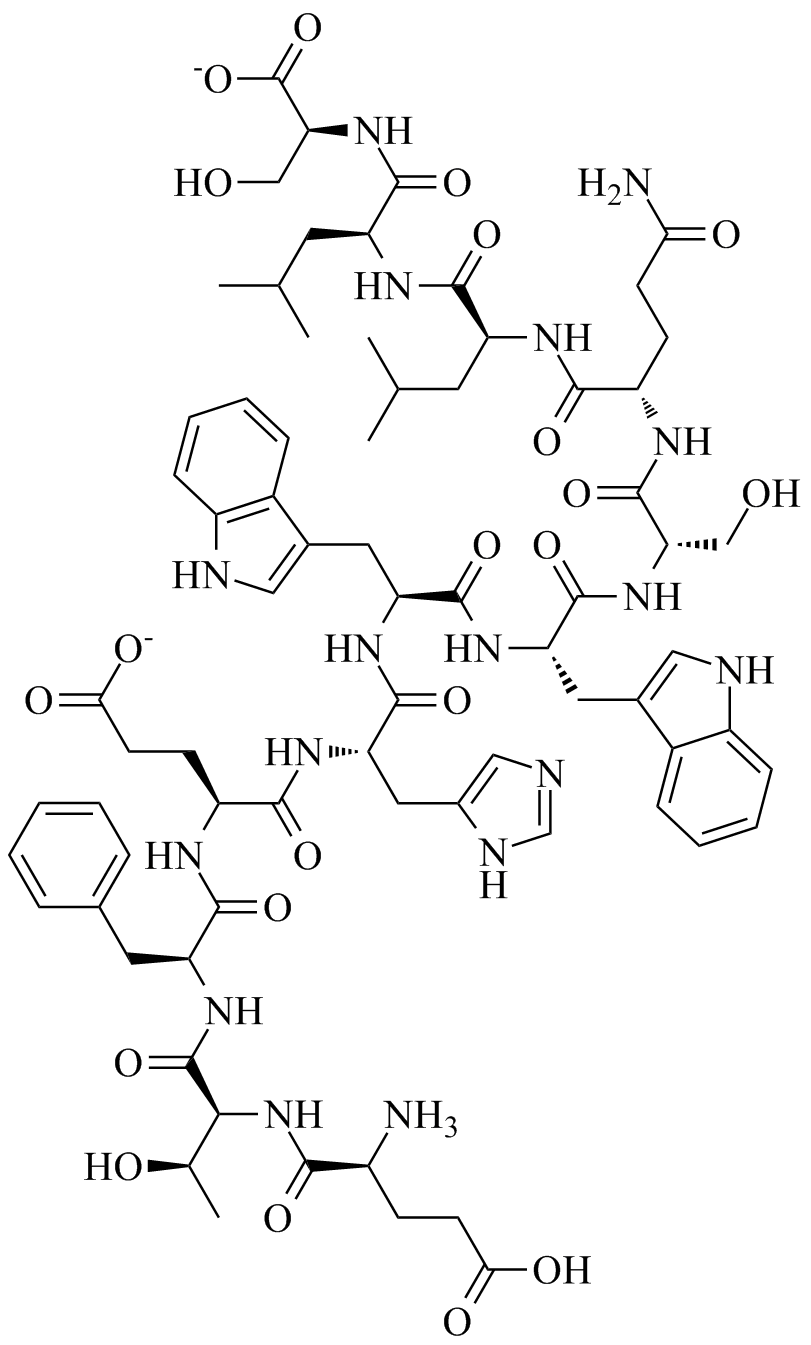  pDIQ | 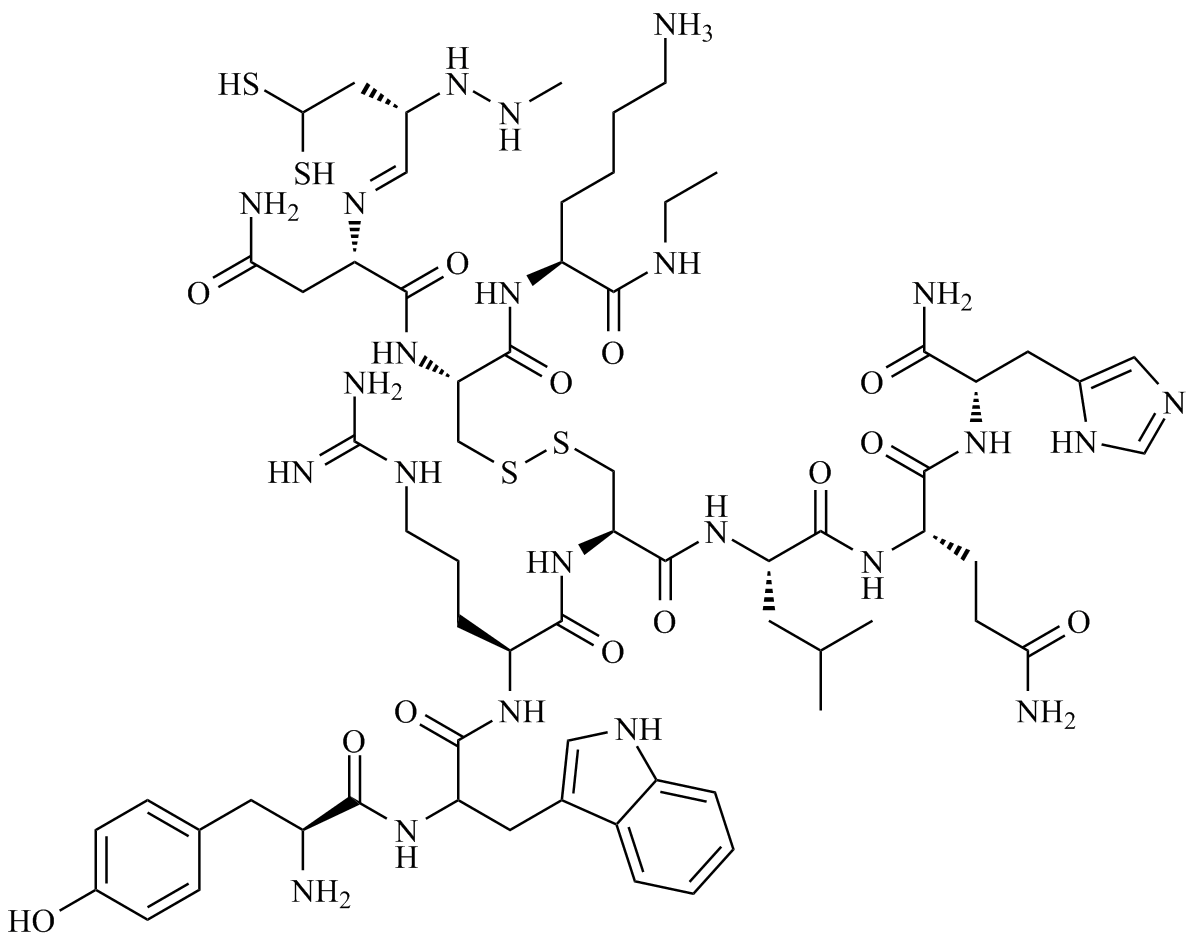  Stingin 1 |  |
