# Supplementary material for: Structure- and Ligand-Based Virtual Screening Identifies New Scaffolds for Inhibitors of the Oncoprotein MDM2
Source: PLoS One. 2015 Apr 17;10(4):e0121424. doi: 10.1371/journal.pone.0121424 (PMC4401541; doi:10.1371/journal.pone.0121424)
Supplement: S2 Table — Rank scores within the various ranking schemes are indicated, noninteger rank scores indicate positions tied between an even number of compounds; positions were determined for all compounds for which Autodock and Vina both predicted similar binding modes; this numbered 2,120 compounds. For those compounds found to be active, the scoring algorithm that ranked them highest is indicated by highlighting in the rank position in yellow. *Compounds found to exhibit activity in both the CE and FP assays. †Compound not in stock at time of acquisition and substituted by the similar compound 39. Results for the known inhibitors MI-63 and Telmisartan are included for comparison. (DOCX) [file pone.0121424.s003.docx]

**S2 Table. Virtual screening statistics of compounds selected for assay.** Rank scores within the various ranking schemes are indicated, noninteger rank scores indicate positions tied between an even number of compounds; positions were determined for all compounds for which Autodock and Vina both predicted similar binding modes; this numbered 2,120 compounds. For those compounds found to be active, the scoring algorithm that ranked them highest is indicated by highlighting in the rank position in yellow. *Compounds found to exhibit activity in both the CE and FP assays. **^†^**Compound not in stock at time of acquisition and substituted by the similar compound **39**. Results for the known inhibitors MI-63 and Telmisartan are included for comparison.

| **Compound ID** | **Supplier** | **Supplier  compound  code** | **Autodock  predicted**  **ΔG (kcal/mol)** | **Autodock rank score** | **Vina  predicted**  **ΔG (kcal/mol)** | **Vina rank score** | **X-Score** | **X-Score rank score** | **DrugScore** | **Drugscore rank score** | **Consensus rank position** |
| --- | --- | --- | --- | --- | --- | --- | --- | --- | --- | --- | --- |
| **Compound 1*** | Asinex | BAS 118474 | -7.42 | 1787.5 | -8.3 | 1552 | 7.48 | 203.5 | -393773 | 1674 | 1494 |
| **Compound 2*** | Asinex | BAS 702133 | -7.79 | 1402 | -8.2 | 1644.5 | 7.61 | 97.5 | -389617 | 1727 | 1316 |
| **Compound 3** | Asinex | BAS 1076265 | -8.22 | 861 | -9.1 | 229.5 | 7.28 | 541.5 | -434382 | 1057 | 361 |
| **Compound 4** | Asinex | BAS 1578940 | -8.34 | 728.5 | -8.1 | 1727 | 7.65 | 75 | -463650 | 616 | 523 |
| **Compound 5** | Asinex | ASN 4455602 | -10.05 | 7 | -8.9 | 452.5 | 7.42 | 281.5 | -549584 | 21 | 45 |
| **Compound 6** | InterBioScreen | STOCK1N-74889 | -8.63 | 446.5 | -9.3 | 113 | 7.1 | 1078 | -439299 | 968 | 334 |
| **Compound 7** | InterBioScreen | STOCK1S-86015 | -7.61 | 1601.5 | -8.4 | 1398.5 | 7.81 | 26.5 | -437309 | 1005 | 913 |
| **Compound 8** | ChemBridge | 21007016 | -8.99 | 208.5 | -8.5 | 1178 | 6.7 | 1907 | -512187 | 135 | 638 |
| **Compound 9** | ChemBridge | 26559956 | -8.92 | 236 | -9.8 | 15 | 7.71 | 50 | -459202 | 674 | 59 |
| **Compound 10** | ChemBridge | 30158441 | -8.73 | 356 | -9.6 | 38.5 | 7.3 | 491 | -516930 | 110 | 62 |
| **Compound 11** | ChemBridge | 33510290 | -8.88 | 263 | -9.6 | 38.5 | 7.41 | 293.5 | -529191 | 65 | 35 |
| **Compound 12** | ChemBridge | 47458824 | -8.2 | 886 | -9 | 331 | 7.04 | 1349 | -500010 | 213 | 398 |
| **Compound 13** | ChemBridge | 60596987 | -8.84 | 293.5 | -9.7 | 25.5 | 7.28 | 541.5 | -489428 | 309 | 84 |
| **Compound 14** | ChemBridge | 69170519 | -8.57 | 506 | -9.3 | 113 | 7.25 | 621.5 | -479870 | 398 | 147 |
| **Compound 15** | ChemBridge | 98566692 | -7.81 | 1382 | -9.3 | 113 | 6.91 | 1613.5 | -376277 | 1867 | 1368 |
| **Compound 16*^†^** | ChemBridge | 5317210 | -7.38 | 1812.5 | -9 | 331 | 7.53 | 153.5 | -383764 | 1788 | 943 |
| **Compound 17** | ChemBridge | 5655839 | -8.12 | 987.5 | -9 | 331 | 7.46 | 226.5 | -438379 | 991 | 316 |
| **Compound 18** | ChemBridge | 9000459 | -7.8 | 1392 | -8.5 | 1178 | 7.44 | 255 | -429566 | 1132 | 872 |
| **Compound 19*** | ChemBridge | 9007617 | -8.37 | 704 | -9 | 331 | 7.48 | 203.5 | -427206 | 1168 | 289 |
| **Compound 20** | LifeChemicals | F0017-0533 | -7.91 | 1269.5 | -9 | 331 | 7.29 | 516.5 | -382995 | 1796 | 846 |
| **Compound 21** | LifeChemicals | F0589-0488 | -8.23 | 851 | -9.2 | 159.5 | 7.36 | 377.5 | -407412 | 1500 | 428 |
| **Compound 22** | LifeChemicals | F0914-3611 | -9.15 | 138 | -9 | 331 | 6.85 | 1721.5 | -413398 | 1393 | 696 |
| **Compound 23** | LifeChemicals | F1057-0391 | -6.93 | 2038 | -9 | 331 | 7.04 | 1349 | -426986 | 1174 | 1324 |
| **Compound 24*** | LifeChemicals | F1352-0058 | -8.44 | 623 | -9 | 331 | 6.69 | 1918.5 | -423197 | 1221 | 946 |
| **Compound 25** | LifeChemicals | F2475-0669 | -7.32 | 1849 | -9 | 331 | 6.55 | 2024.5 | -416864 | 1335 | 1641 |
| **Compound 26** | LifeChemicals | F2477-0068 | -8.71 | 371.5 | -9.2 | 159.5 | 6.86 | 1705.5 | -441772 | 925 | 533 |
| **Compound 27** | LifeChemicals | F2707-0011 | -8.65 | 428 | -9.4 | 78.5 | 7.09 | 1124 | -415946 | 1347 | 453 |
| **Compound 28** | Specs | AN-153/I100227 | -7.38 | 1812.5 | -9 | 331 | 7.15 | 906 | -361414 | 1974 | 1396 |
| **Compound 29** | Specs | AN-153/I257031 | -6.81 | 2061.5 | -8.9 | 452.5 | 7.63 | 86.5 | -402272 | 1572 | 981 |
| **Compound 30** | ChemDiv | 3473-1206 | -8.34 | 728.5 | -9 | 331 | 7.75 | 37.5 | -456471 | 707 | 177 |
| **Compound 31** | ChemDiv | 3473-1209 | -8.43 | 635.5 | -8.5 | 1178 | 7.68 | 61 | -471253 | 509 | 287 |
| **Compound 32** | ChemDiv | D077-0309 | -9.24 | 114 | -9.1 | 229.5 | 7.48 | 203.5 | -394518 | 1664 | 258 |
| **Compound 33** | ChemDiv | G243-1709 | -9.05 | 175.5 | -8 | 1808 | 7.1 | 1078 | -409403 | 1471 | 1161 |
| **Compound 34** | Enamine | T5259122 | -10.07 | 6 | -8.9 | 452.5 | 7.49 | 194 | -514719 | 122 | 46 |
| **Compound 35** | Enamine | T5616586 | -9.34 | 82 | -9 | 331 | 7.62 | 91.5 | -538265 | 39 | 21 |
| **Compound 36** | Enamine | T0504-1590 | -7.94 | 1224 | -8.6 | 960 | 7.66 | 70 | -395724 | 1651 | 841 |
| **Compound 37** | Enamine | T6114030 | -8.19 | 897.5 | -9.2 | 159.5 | 7.3 | 491 | -400646 | 1590 | 517 |
| **Compound 38** | Enamine | T5383638 | -7.6 | 1611.5 | -8.7 | 764 | 7.43 | 268 | -432028 | 1097 | 769 |
| **Telmisartan** | - | - | -10.66 | 1 | -9.9 | 7 | 8.75 | 1 | -665276 | 1 | 1 |
| **MI-63** | - | - | -9.50 | 52.5 | -9.9 | 7 | 7.96 | 4.5 | -423936 | 1210 | 99 |
