# Supplementary material for: Structure- and Ligand-Based Virtual Screening Identifies New Scaffolds for Inhibitors of the Oncoprotein MDM2
Source: PLoS One. 2015 Apr 17;10(4):e0121424. doi: 10.1371/journal.pone.0121424 (PMC4401541; doi:10.1371/journal.pone.0121424)
Supplement: S3 Table — *Molecules that passed the "consensus docking" filter criteria. †Molecules for which Autodock finds only one cluster of docking solutions—experience suggests that these tend to be more reliable predictions of binding mode. (DOCX) [file pone.0121424.s004.docx]

**S3 Table. Summary of docking energies of fragment low energy binding modes versus controls.** *Molecules that passed the "consensus docking" filter criteria. ^†^Molecules for which Autodock finds only one cluster of docking solutions - experience suggests that these tend to be more reliable predictions of binding mode.

| Compound | Vina cluster (out of 9) that  matches Autodock pose | Vina ΔG (kcal/mol) | Vina ΔG best/worst (kcal/mol) | Autodock ΔG (kcal/mol) | Number of  Autodock clusters | Autodock cluster  ΔG best/worst (kcal/mol) |
| --- | --- | --- | --- | --- | --- | --- |
| 40 | 3rd | -5.1 | -5.3/-4.8 | -4.6 | 1^†^ | -4.6/-4.6 |
| 41 | 2nd | -6.6 | -6.7/-5.9 | -5.7 | 3 | -5.7/-5.5 |
| 42 | 3rd | -5.8 | -6.2/-5.4 | -5.4 | 5 | -5.4/-4.9 |
| 43 | 1st* | -6.8 | -6.8/-5.5 | -5.4 | 1^†^ | -5.4/-5.4 |
| 44 | 5th | -5.8 | -6.1/-5.5 | -5.3 | 4 | -5.3/-4.7 |
| 45 | 1st* | -7.2 | -7.2/-5.8 | -5.8 | 5 | -5.8/-4.9 |
| MI-63 | 1st* | -9.9 | -9.9/-7.1 | -9.5 | 1^†^ | -9.5/-9.5 |
| Telmisartan | 4th | -9.9 | -9.9/-8.5 | -10.7 | 3 | -10.7/-9.1 |
