# Supplementary material for: Structure- and Ligand-Based Virtual Screening Identifies New Scaffolds for Inhibitors of the Oncoprotein MDM2
Source: PLoS One. 2015 Apr 17;10(4):e0121424. doi: 10.1371/journal.pone.0121424 (PMC4401541; doi:10.1371/journal.pone.0121424)
Supplement: S4 Table — Larger molecules (with the exception of the flexible Telmisartan) tend to exhibit clearer solutions than fragments. (DOCX) [file pone.0121424.s005.docx]

**S4 Table. Vina predicted ΔG for each of its top 9 docking solutions.** Larger molecules (with the exception of the flexible Telmisartan) tend to exhibit clearer solutions than fragments.

| 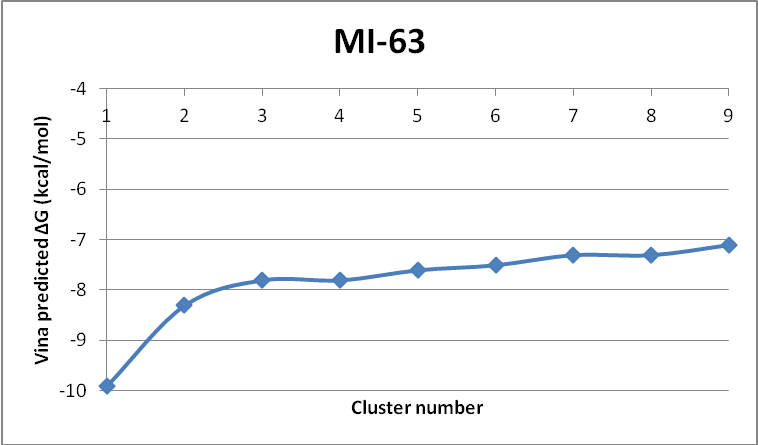 | 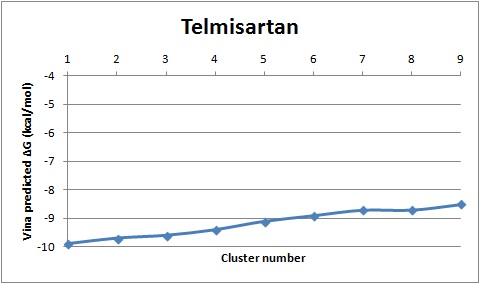 |  |
| --- | --- | --- |
|  |  |  |
|  |  |  |
|  |  |  |
