# Supplementary material for: Structure- and Ligand-Based Virtual Screening Identifies New Scaffolds for Inhibitors of the Oncoprotein MDM2
Source: PLoS One. 2015 Apr 17;10(4):e0121424. doi: 10.1371/journal.pone.0121424 (PMC4401541; doi:10.1371/journal.pone.0121424)
Supplement: S5 Table — (DOCX) [file pone.0121424.s006.docx]

**S5 Table. Summary of the solubilities of the fragments in assay buffer.**

| **Compound** | **Structure** | **Estimated solubility limit** |
| --- | --- | --- |
| 40 |  | >2 mM |
| 41 |  | 0.25 mM to 0.5 mM |
| 42 |  | 0.25 mM to 0.5 mM |
| 43 |  | 0.25 mM to 0.5 mM |
| 44 |  | 0.0625 mM to 0.125 mM |
| 45 |  | >1m M |
| 46 |  | >1m M |
